# Canal-wall up cholesteatoma surgery with mastoid obliteration leads to lower rates of disease recurrence without affecting hearing outcomes

**DOI:** 10.3389/fsurg.2024.1381481

**Published:** 2024-04-08

**Authors:** Chiara Erfurt, Sanne F. Westerhout, Louise V. Straatman, Adriana L. Smit, Robert J. Stokroos, Hans G. X. M. Thomeer

**Affiliations:** ^1^ Department of Otorhinolaryngology and Head & Neck Surgery, University Medical Center Utrecht, Utrecht, Netherlands; ^2^ Brain Center, University Medical Center Utrecht, Utrecht, Netherlands

**Keywords:** cholesteatoma, recurrent disease, tympanomastoidectomy, mastoid obliteration, hearing loss, middle ear disease, canal-wall up (CWU)

## Abstract

**Objectives:**

The primary objective was to determine whether obliteration of the epitympanic area and mastoid cavity during canal wall up (CWU) cholesteatoma surgery reduces the rate of recurrent and residual cholesteatoma compared to not obliterating the same area. The secondary objective was to compare postoperative hearing outcomes between both techniques.

**Methods:**

A retrospective cohort study was conducted in a tertiary referral center. One-hundred-fourty-three ears were included of patients (≥18y) who underwent a CWU tympanomastoidectomy for cholesteatoma with or without bony obliteration between January 2015 and March 2020 in the University Medical Center Utrecht. The median follow-up was respectively 1.4 (IQR 1.1–2.2) vs. 2.0 years (IQR 1.2–3.1) (*p* = 0.013).

**Intervention(s):**

All patients underwent CWU tympanomastoidectomy for cholesteatoma. For 73 ears bone dust, Bonalive® or a combination was used for obliteration of the mastoid and epitympanic area, the rest of the ears (*n* = 70) were not obliterated. In accordance with the Dutch protocol, included patients are planned to undergo an MRI scan with diffusion-weighted imaging (DWI) one, three and five years after surgery to detect recurrent or residual cholesteatoma.

**Main outcome measure(s):**

The primary outcome measure was recurrent and residual cholesteatoma as evaluated by MRI-DWI and/or micro-otoscopy and confirmed by micro-otoscopy and/or revision surgery. The secondary outcome measure was the postoperative hearing.

**Results:**

In this cohort, the group treated with canal wall up tympanomastoidectomy with subsequent bony obliteration (73 ears, 51.0%) had significantly lower recurrent (4.1%) and residual (6.8%) cholesteatoma rates than the group without obliteration (70 ears, 25.7% and 20.0%, respectively; *p* < 0.001). There was no significant difference between both groups in postoperative bone conduction thresholds (mean difference 2.7 dB, *p* = 0.221) as well as the mean air-bone gap closure 6 weeks after surgery (2.3 dB in the non-obliteration and 1.5 dB in the obliteration group, *p* = 0.903).

**Conclusions:**

Based on our results, a canal wall up tympanomastoidectomy with bony obliteration is the treatment of choice, since the recurrent and residual disease rate is lower compared to the group without obliteration. The bony obliteration technique does not seem to affect the perceptive or conductive hearing results, as these are similar between both groups.

## 1 Introduction

The goal of cholesteatoma treatment is to eradicate the disease and create a safe and dry ear, while preventing recurrence of pathology and preserving or even optimizing hearing (1). The best surgical approach for treatment of cholesteatoma has been subject to debate for decades. Although canal wall down (CWD) and canal wall up (CWU) tympanomastoidectomy both have their advantages and disadvantages, the CWU technique is often preferred since it avoids creating a radical cavity. A radical cavity might provide a situation with higher rates of recurrent otitis and requires regular debridement. In contrast, the CWU technique maintains the normal anatomy of the external ear canal, generally resulting in fewer cases of refractory otitis. Moreover, after CWU surgery regular debridement of the ear is generally not indicated and hearing aids fit better and are better tolerated (2–4). However, a significant concern associated with opting for the CWU approach is the increased likelihood of recurrent and residual cholesteatoma (5). In previous literature, rates of recidivism—including both recurrent and residual rates—have been found to be 4 to 17% by using CWD tympanomastoidectomy compared to rates between 9% and 70% by using the CWU approach (5, 6). Since the large range in prevalence cannot be explained only by the surgical approach, there must be numerous other factors that influence the variety, such as experience of the surgeon and patient related factors.

There has been growing interest in obliterating the epitympanic and mastoid area subsequent to either CWD or CWU tympanomastoidectomy, with the primary goal of optimizing disease control. The first attempts were published in 1911 by Mosher (7). Mercke was one of the first otologists to present outcomes with low recurrent and residual rates after obliteration in combination with CWD tympanomastoidectomy (8). Promising results as presented by Offeciers et al. followed with a different technique, leaving the posterior canal wall intact (9). Recently, van der Toom et al. showed in their systematic review that obliterating the mastoid reduces the recidivism rates for both the CWD (5.9% recurrent rate, 5.8% residual rate) and CWU approach (0.28% recurrent rate, 4.2% residual rate) (6). Combining the CWU approach with obliteration therefore seems to be a good solution to optimize disease control.

Hearing is another crucial outcome of cholesteatoma surgery. The available research that focuses on hearing outcomes has predominantly examined the CWD approach in combination with the bony obliteration technique (BOT). In the literature this procedure is described either with or without canal wall reconstruction. Numerous studies have shown that by using the combination of these two techniques (CWD with BOT), preoperative hearing outcomes are preserved or even moderately improve after surgery (10–13). There are only a few available studies on hearing outcomes after CWU surgery with BOT. Nonetheless, they do show promising results regarding hearing preservation (14–17). It is important to note that during the obliteration of the mastoid and epitympanum, a part of the malleus as well as the incus are removed, which can influence postoperative hearing. Further research must address this aspect in order to be able to draw definitive conclusions.

Since CWU cholesteatoma surgery has been commonly performed in our tertiary center for years, with introduction of the BOT as an addition to this approach in 2015, our cohort of patients is very suitable to compare both surgical techniques.

The primary aim of this study was to determine whether CWU tympanomastoidectomy with bony obliteration improves disease control of cholesteatoma compared to CWU surgery without obliteration. Secondly, postoperative hearing outcome was evaluated and compared between the two mentioned techniques.

## 2 Materials and methods

A retrospective cohort study was performed in our tertiary referral center with approval of the medical research ethical committee (MvdL/mb/21/500229). Patients, above the age of 18, who underwent primary or revision CWU tympanomastoidectomy, both with or without bony obliteration of the epitympanic area and mastoid cavity for treatment of their cholesteatoma, were identified. All surgeries were performed by four surgeons in the University Medical Center (UMC) Utrecht between January 2015 and March 2020. Patients were included when evaluated for recurrent and residual disease at least once, around one year after surgery, by magnetic resonance imaging with diffusion-weighted imaging (MRI-DWI) and by micro-otoscopy.

When diffusion restriction indicating cholesteatoma was seen on MRI-DWI during follow-up, a revision surgery was performed. When no cholesteatoma was found during this surgery the MRI-DWI was marked as false-positive. Sometimes it was not possible to give a definitive answer on the occurrence of residual or recurrent cholesteatoma based on the diffusion restriction. In that case the result was described as “questionable”, revision surgery was postponed and the MRI-DWI was repeated one year afterwards. Occasionally, a revision surgery was performed directly without making an MRI, due to specific reasons such as evident recurrent disease present during micro-otoscopy. Recurrent disease encompasses a newly formed retraction pocket with cholesteatoma, visible by either micro-otoscopy, detected on MRI-DWI or during second look surgery (10, 18). Residual disease consists of epithelium (cholesteatoma matrix) left behind during surgery, suspected on MRI-DWI and confirmed during surgery. Patients aged under 18 at the time of surgery were excluded, as well as cases regarding radical cavities or other surgical techniques than those compared (e.g., endoscopic or endaural). Also, cases in which the surgeon deliberately had left epithelium in the surgical field, attached to delicate structures (i.e., membranous labyrinth, dura mater, facial nerve), were excluded.

Besides the main outcome measures, recidivism and hearing outcomes, we also investigated any differences in patient and surgical characteristics. These included, sex, age, side (left/right), comorbidities, number of primary cases, obliteration material, type of ossicular chain reconstruction, extent of the cholesteatoma according to the STAMCO classification, complications attributable to the extent of the cholesteatoma (labyrinth fistula, tegmen defect and facial nerve dehiscence), surgical complications and duration of surgery (19). Follow-up time was defined as the maximum time between surgery and the most recent outpatient clinic visit. The last possible follow-up date within this cohort was set at April 30th 2021, allowing sufficient time for conducting the MRI-DWI one year after the last patient underwent surgery. Disease control was expressed in a rate from 0 to 1.0, in which 0 equals 100% and 1.0 equals 0% of recurrent and residual disease. The recidivism rates were also analysed individually for the STAMCO stages (I, II, III and IV) to identify possible differences depending on location and disease extension. The following classification was used, in accordance with the EAONO/JOS Joint Consensus Statements; stage I: Cholesteatoma localized in the primary site, stage II: Cholesteatoma involving two or more sites, stage III: Cholesteatoma with extracranial complications or pathologic conditions, stage IV: Cholesteatoma with intracranial complications (20). As it is the aim of this study to determine whether there is a difference in outcome between the two techniques, each surgery, rather than each patient, was evaluated as a separate case. All necessary parameters were transmitted from the electronic patient record system to an anonymized file by the datamanager of the UMC Utrecht surgical department. When required information was missing, the specific patient file was assessed in detail by the principal investigator. When a variable was not recorded in the electronic patient dossier, it was classified as “missing data”.

### 2.1 Hearing outcome

Hearing was assessed approximately four weeks preoperatively by pure-tone audiometry, as well as six weeks postoperatively. The pure tone average thresholds (PTA) were calculated for air and bone conduction (AC, BC respectively) at 0.5, 1, 2, and 4 kHz (21). Afterwards the air-bone gap was calculated. The Amsterdam Hearing Evaluation Plot (AHEP) was used to visualize the pre- and postoperative bone conduction and box plots were used to visualize the mean air-bone gap closure.

### 2.2 Surgical technique

To perform a CWU procedure, a retroauricular incision was applied (cutis and subcutis). Then an anterior pedicled musculoperiostal flap was elevated and lateralized; a posterior incision in the canal skin was made and the tympanic membrane was inspected. A tympanomeatal flap was created and middle ear work was performed; assessing the ossicular chain, visualizing routing of the cholesteatoma, determining whether it was either a recurrent or residual cholesteatoma and cleaning of the middle ear. A mastoidectomy, atticoantrotomy was performed with preservation of the posterior bony canal wall. All of the cholesteatoma was removed respecting the critical landmarks (labyrinth, facial nerve, tegmen, middle fossa bony plate, sigmoid sinus). A combined approach technique was used to ascertain the surgeon that all pathology was removed (when necessary including a posterior tympanotomy, also known as facial recess approach). For bony obliteration a bone chip and cortical bone dust was harvested from the mastoid cortex using a drill with 10.000–20.000 revolutions per minute. Both the bone chip and bone dust were preserved in povidon iodine 10% aqueous solution. These were used to close off the epitympanic space from the middle ear and to fill up the epitympanic space and mastoid at the final stage of the procedure. Fibrin glue (Tisseel®, Baxter) was applied to keep the reconstruction in place. In some cases bioactive glass granules (Bonalive®) were used instead of or in addition to the bone dust. At our tertiary center we started implemented the obliteration technique alongside the traditional technique in 2015. All surgeons participating in this study utilized both techniques. The reconstruction of the tympanic membrane was performed using autologous material (temporalis fascia, conchal cartilage, perichondrium) and the ossicular chain reconstruction was performed using allogenous material (titanium ossicular reconstruction, KurzMedical®) or by means of a type III tympanoplasty. We refer to a type III tympanoplasty when a tympanic membrane graft was placed directly onto the stapes head. Lastly, skin closure was performed in three layers, the meatus was dressed with gauze soaked in antibiotic ointment (for 7–10 days) and a pressure head bandage was applied for 24 h. Patients of the obliteration group received 625 mg Augmentin 3 times a day for 5 days postoperatively.

### 2.3 Statistical analysis

Statistical analysis was performed using the software platform “Statistical Package for the Social Sciences” 29.0.1 (SPSS Inc., Chicago, IL, USA). Baseline characteristics per group were described with means and standard deviations or medians and interquartile ranges in Table 1 depending on normality. To compare the difference in recurrent and residual disease between both surgical groups and between all STAMCO stages, the chi square test was used. Due to not normally distributed data, the Mann-Whitney U test was performed to compare the audiological data and the follow-up time between the two groups. The same test was used to assess the difference in air-bone gap closure between all STAMCO stages. Descriptive statistics using frequencies and means were used to summarize the remaining research data. A significant result was defined as a *p*-value <0.05.

**Table 1:** Patient characteristics per surgical technique.

	Non-obliteration	Obliteration	All ears
Total number of ears	70 (49.0%)	73 (51.0%)	143 (100%)
Mean age (range)	43 (18–84)	44 (19–76)	43.5 (18–84)
Median follow-up years (IQR)*	2.0 (1.2–3.1)	1.4 (1.1–2.2)	1.8 (1.1–2.6)
Sex			
Male	41 (58.6%)	47 (64.4%)	88 (61.5%)
Female	29 (41.4%)	26 (35.6%)	55 (38.5%)
Side			
Left	38 (54.3%)	41 (56.2%)	79 (55.2%)
Right	32 (45.7%)	32 (43.8%)	64 (44.8%)
Primary surgery**	32 (45.7%)	14 (19.2%)	46 (32.2%)
Median duration of surgery in minutes (IQR)	151 (128–186)	157 (131–187)	154 (130–187)
Extent/location of cholesteatoma (STAMCO)			
Stage I	3 (4.3%)	8 (11.0%)	11 (7.7%)
Stage II	34 (48.6%)	26 (35.6%)	60 (42.0%)
Stage III	33 (47.1%)	39 (53.4%)	72 (50.3%)
Stage IV	0	0	0
Ossicular chain reconstruction			
Chain intact	8 (11.4%)	1 (1.4%)	9 (6.3%)
No reconstruction during surgery	5 (7.1%)	11 (15.1%)	16 (11.2%)
PORP/TORP already *in situ*	2 (2.9%)	8 (11.0%)	10 (7.0%)
Tympanoplasty type III	14 (20.0%)	15 (20.5%)	29 (20.3%)
PORP	27 (38.6%)	24 (32.9%)	51 (35.7%)
TORP	14 (20.0%)	14 (19.2%)	28 (19.6%)
Complications due to cholesteatoma			
Labyrinth fistula	6 (8.6%)	7 (9.6%)	13 (9.1%)
Tegmen defect	21 (30.0%)	15 (20.5%)	36 (25.2%)
Facial nerve dehiscence	13 (18.6%)	19 (26.0%)	32 (22.4%)
Obliteration material			
Autologous bone	–	58 (79.5%)	–
Bonalive	–	9 (12.3%)	–
Combination autologous bone and Bonalive	–	6 (8.2%)	–
Comorbidities			
Diabetes mellitus type 2	3 (4.3%)	1 (1.4%)	4 (2.8%)
COPD	5 (7.1%)	2 (2.7%)	7 (4.9%)
Asthma	7 (10.0%)	8 (11.0%)	15 (10.5%)
Atopic constitution	7 (10.0%)	10 (13.7%)	17 (11.9%)
Chronic rhinosinusitis	10 (14.3%)	6 (8.2%)	16 (11.2%)
Smoking	21 (30%)	30 (41.1%)	51 (35.7%)

PORP, partial ossicular reconstruction prosthesis; TORP, total ossicular reconstruction prosthesis. Asterisks indicates a statistically significant difference between groups.

* *p* < 0.05.

** *p* = 0.001.

## 3 Results

### 3.1 Demographics

143 ears in 112 patients were treated within the period between January 2015 and March 2020. In 70 ears no obliteration and in 73 ears a bony obliteration was performed. The median follow-up was respectively 2.0 (IQR 1.2–3.1) vs. 1.4 years (IQR 1.1–2.2) (*p* = 0.013). The median time between the surgery and the first MRI-DWI did not differ significantly between the two groups (0.98 years for the non-obliteration group, 1.02 years for the obliteration group, *p* = 0.159). There were 32 out of 70 (45.7%) primary surgeries in the non-obliteration vs. 14 out of 73 (19.2%) in the obliteration group (*p* = 0.001). There were no significant differences in other demographic characteristics between both groups (Table 1). The median duration of surgery was 151 min (IQR 128–186; non-obliteration) vs. 157 min (IQR 131–187; obliteration). There were 6 out of 70 ears (non-obliteration) and 7 out of 73 (obliteration group) ears with a labyrinthine fistula. The ossicular chain was intact in 8 cases of the non-obliteration group and in 1 of the obliteration group. A PORP was placed in 27 and 24 cases respectively and a TORP in 14 cases in both groups. The rest of the cases either already had a prothesis *in situ*, no reconstruction was performed or a tympanoplasty type III was performed. One patient in the non-obliteration group (1.4%) and two in the obliteration group (2.7%) developed a post-operative wound infection and were treated with antibiotics. One patient in the obliteration group was admitted due to a post-operative headache which developed one day after surgery. This patient had a large cholesteatoma which expanded to the labyrinth as well as chronic otitis media which was already being treated with oral antibiotics. This was switched to intravenous antibiotics and the headache resolved after a few days.

### 3.2 Disease control

**Figure 1:**
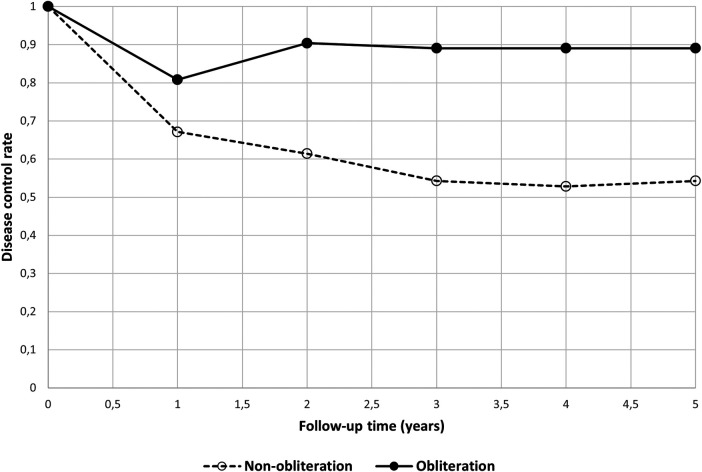
Disease control rate by surgical technique: non-obliteration versus obliteration group. Striped open line represents the group without obliteration; black line represents the group with obliteration. On the x-axis the follow-up time in years, on the y-axis the disease control rate (a disease control rate of 1 represents 0% recurrent or residual cholesteatoma cases).

The group without bony obliteration showed an overall recidivism rate of 45.7% (32 out of 70) with recurrent cholesteatoma in 18 ears (25.7%) and residual disease in 14 ears (20.0%). The overall recidivism rate for the group with bony obliteration was 11.0% (8 out of 73), of which 3 (4.1%) were recurrent and 5 (6.8%) residual cholesteatomas. The difference between both surgical groups was statistically significant (*p* < 0.001). Almost all recurrent and residual cases had previous ear surgery (20/32 non-obliteration; 8/8 obliteration). At 6 weeks no cholesteatoma was seen during micro-otoscopy and after 12 months one cholesteatoma was identified by micro-otoscopy. This case also showed diffusion restriction on the MRI-DWI. One patient in the non-obliteration group underwent an MRI between 6 weeks and 12 months due to symptoms of hearing loss. The MRI of this patient showed signs of residual disease and a revision surgery was performed. In two cases, both belonging to the non-obliteration group, a second-look surgery was performed without an MRI within the first year after surgery. In the first case, surgery was conducted due to chronic inflammation and inadequate hearing rehabilitation. In the second case, it was performed for evaluation of the middle ear and an ossicular chain reconstruction in a case with a previously reconstructed fistula of the lateral semicircular canal. During the second-look surgery of the latter case, residual disease was detected. After one year, the rate of disease control was 0.67 for the non-obliteration group and 0.81 for the obliteration group (Figure 1). After two years, the difference had increased with a rate of 0.61 and 0.90, respectively. After three years the rates were 0.54 and 0.89.

### 3.3 STAMCO

In both the non-obliteration and obliteration group most of the cases were staged during the original surgery as STAMCO stage II (non-obliteration *n* = 34, obliteration *n* = 26) and III (non-obliteration *n* = 33, obliteration *n* = 39). The recidivism rates between the two techniques did not significantly differ for stage I (non-obliteration 25.0% vs. obliteration 14.3%, *p* = 0.308) and stage II (non-obliteration 38.2% vs. obliteration 15.4%, *p* = 0.122). A significant difference was found for stage III (non-obliteration 54.5% vs. obliteration 7.7%, *p* < 0.001). There were no cases classified as stage IV (Supplementary File S1).

### 3.4 Hearing outcome

**Figure 2:**
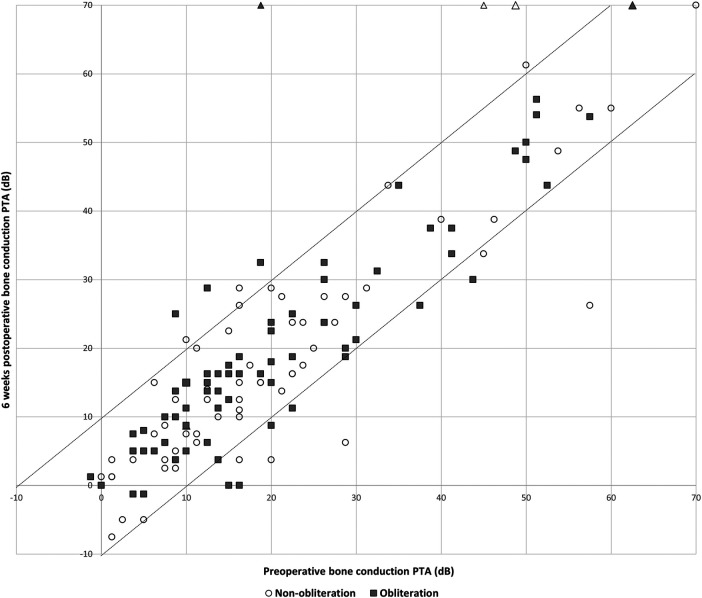
Amsterdam hearing evaluation plot, *n* = 138: individual cases of preoperative bone conduction pure tone average against 6 weeks postoperative bone conduction pure tone average. The postoperative BC PTA is plotted against the preoperative BC PTA. Dots below the lower dotted diagonal line represent cases where the BC PTA improved more than 10 dB. Dots above the upper diagonal line represent ears with a decrease in the postoperative BC PTA threshold more than 10 dB. When a dot is located between the two diagonal lines, BC PTA did not change over more than 10 dB. The open circles represent cases of the non-obliteration group; the filled squares represent cases of the obliteration group. Triangles represent cases with postoperative functional deafness.

**Figure 3:**
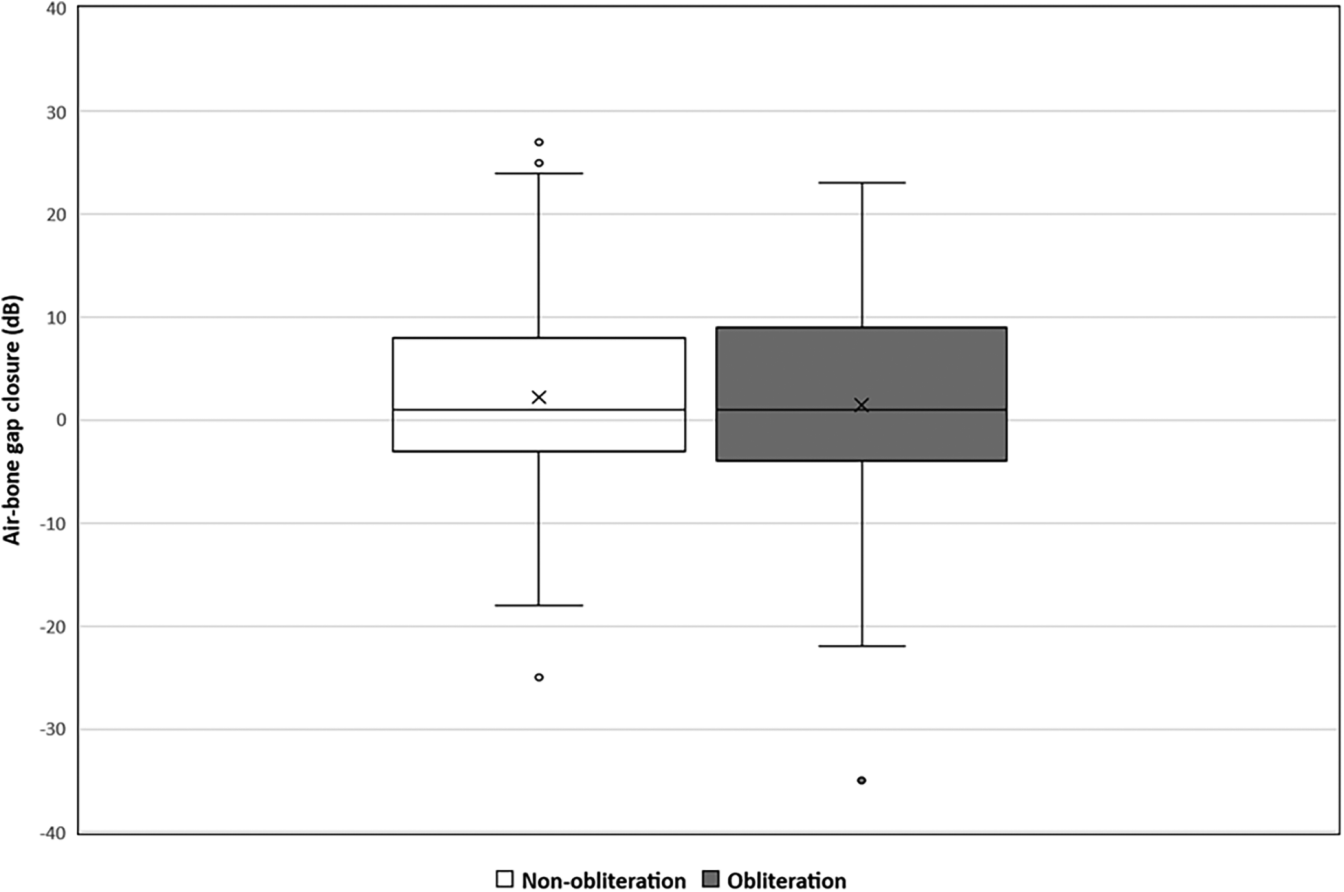
Mean air-bone gap closure in decibels divided by surgical approach: non-obliteration (*n* = 67) versus obliteration group (*n* = 71). The X in each box represent the mean. The dots represent the outliers. The mean air-bone gap closure 6 weeks postoperative in the non-obliteration group was 2.3 dB and 1.5 dB in the obliteration group (mean difference 0.7 dB, 95% CI 0.05–3.63, *p* = 0.903).

Pre-operative hearing tests were available for all cases except one from the non-obliteration group. Post-operative hearing tests after 6 weeks were available for 67/70 (95.7%) in the non-obliteration group and 71/73 (97.2%) cases in the obliteration group. There was no significant difference found in AC threshold, BC threshold and ABG between the two groups. The median pre-operative BC PTA threshold was 16.0 dB in both the non-obliteration and obliteration group (IQR 9–24 and 9–29 respectively) (Table 2). The median BC PTA threshold post-operatively was 14.0 dB in the non-obliteration group and 15.1 dB in the obliteration group (IQR 6–24 and 8–29 respectively). To give a visual representation of the sensorineural hearing results of each individual case an Amsterdam Hearing Evaluation Plot was plotted (Figure 2) (22). In 3 out of 70 (4.2%) non-obliterated and 3 out of 73 (4.2%) obliterated ears the BC PTA threshold deteriorated more than 10 dB after surgery (Figure 2). Moreover, in both the non-obliteration and obliteration group two cases acquired functional deafness after surgery (indicated by triangles in Figure 2): in the first group the causes were unclear, in the latter one patient had a destructive labyrinthine fistula (stage III) and the other had an attack attributed to the previously diagnosed Meniere's disease. One patient in the non-obliteration group had pre-existing total deafness. The mean ABG closure 6 weeks postoperatively was 2.3 dB in the non-obliteration and 1.5 dB in the obliteration group (*p* = 0.903, Figure 3). When cases were subdivided by STAMCO stage, there was no difference in ABG closure in any of the stages (Supplementary File S2).

**Table 2:** Hearing outcome per surgical technique.

		Air-conduction (dB)			Bone-conduction (dB)			Air-bone gap (dB)	
		Pre-operative	Post-operative^a^	Change	Pre-operative	Post-operative^a^	Change	Pre-operative	Post-operative^a^
Obliteration	Mean (SD)	49.5 (19.7)	46.9 (20.1)	2.5	20.6 (14.8)	19.5 (15.1)	1.1	28.9 (10.84)	27.4 (10.2)
	Median (IQR)	46.0 (35–59)	44.0 (29–59)	2.0	16.0 (9–29)	15.1 (8–29)	0.9	28.0 (20–37)	26.0 (20–35)
Non-obliteration	Mean (SD)	48.2 (20.1)	44.0 (20.3)	4.2	18.7 (14.5)	16.8 (14.7)	1.9	29.5 (11.45)	27.2 (9.1)
	Median (IQR)	45.0 (33–60)	38.0 (28–60)	7.0	16.0 (9–24)	14.0 (6–24)	2.0	31.0 (21–38)	25.0 (20–34)

Key: dB, decibel; SD, standard deviation; IQR, interquartile range.

^a^ After 6 weeks.

### 3.5 MRI-DWI follow-up

Twenty-three out of 70 non-obliterated ears (32.9%) showed diffusion restriction on their first MRI-DWI during follow-up, compared to 14 out of 73 obliterated ears (19.2%). Four of the 23 turned out to be false-positive: three underwent a revision surgery during which no cholesteatoma was found, in one diffusion restriction disappeared on the second MRI. In the obliteration group, 2 out of 14 cases were false-positive: diffusion restriction disappeared on the second MRI. In 3 out of 23 (non-obliteration) and 5 out of 14 (obliteration) diffusion restriction was doubtful: it did not disappear over the years, but it also did not grow. Moreover, these patients did not have any complaints and no additional surgery was necessary.

Among the non-obliterated ears, 16 out of 70 (22.9%) exhibited no diffusion restriction on the initial MRI after surgery but later showed recidivism, compared to only 1 out of 73 (1.4%) obliterated ears. These cases either showed diffusion restriction on a second MRI or cholesteatoma was seen during micro-otoscopy or during second look surgery. In the non-obliteration group 12/16 cases were recurrent and 4/16 residual cholesteatomas. The one case in the obliteration group was a recurrent cholesteatoma.

## 4 Discussion

### 4.1 Disease control

Our results demonstrate an overall recidivism rate of 11.0% after bony obliteration compared to 45.7% without obliteration (*p* < 0.001). In 4.1% of the obliteration cases in our study a recurrent cholesteatoma arose, which was significantly lower than in the non-obliteration group (25.7%). This is in line with other recent studies (Table 3). The relatively high residual rates for CWU surgery in previous studies are usually attributed to inadequate overview of the areas affected by cholesteatoma (24–27). Consequently, not all epithelium might be eradicated. This could also attribute to the cases with residual disease within our study in the obliteration as well as the non-obliteration group, but does not explain the difference in outcomes between both groups. Previously, it is suggested that obliterating the cavity reduces the exposure of diseased mucosa, which has suboptimal aeration and gas exchange. A smaller middle ear cavity is created, preventing future tympanic membrane retractions and therefore decreasing the occurrence of recurrent disease (6, 8, 14, 15, 17, 24, 28, 29).

**Table 3:** Study outcomes and characteristics of the bony obliteration technique of four other recent comparable studies.

Study	Recurrence	Residual	Patients (*n*)	Surgery (primary; revision)	Type of obliteration material	Extent of cholesteatoma	Labyrinthine fistula	Median surgery time
van Dinther et al. (14)	2.9%	5.8%	Children with acquired cholesteatoma (34)	50%; 50%	Bone pâté 100%	No information	Not available	Not available
Hellingman et al. (15)	7.1%	7.1%	Adults (74) Children (24)	26%; 74%	Bone pâté and/or hydroxyapatite (no percentages available)	Primary cases: Epitympanic cholesteatomas with 85% extension to middle ear and 81% to antrum and/or mastoid. Revision cases: No information.	Not available	Not available
van der Toom et al. (23)	4.4%	3.2%	Adults (208)	34%; 66%^a^	Bone pâté 79% Bonalive® 1% Combination 20%	STAMCO Stage I 20%; Stage II 70%; Stage III 9%; Stage IV 0%	Not available	318 min
Westerberg et al. (16)	8.0%	1.0%	>12 years (157) <12 years (73)	100%; 0%	Cartilage and cortical bone chips 100%	No information	10 cases (4.3%)	Not available
Current study	4.1%	6.8%	Adults (73)	29%; 81%	Bone dust 80% Bonalive® 12% Combination 8%	STAMCO Stage I 11%; Stage II 36%; Stage III 53% Stage IV 0%	7 cases (9.6%)	157 min

^a^ Distribution of primary versus revision surgeries is not individually reported for the bony obliteration group; this is the distribution within all patients.

In our cohort most of the cases were graded as a STAMCO stage III, indicating extra-cranial complications or pathologic conditions caused by an extensive cholesteatoma. It must be taken into account that in our tertiary center smaller cholesteatomas (STAMCO I and II) are often operated endoscopically or via an endaural approach and as a result are excluded from this study. This is in contrast to van der Toom et al., where a more even distribution between STAMCO I, II and III cholesteatomas was evaluated (23).

Other publications provide similar results of recidivism rate but mostly do not clearly describe the extent of their included cholesteatomas. Furthermore, in 9.1% (13 out of 143) of all cases of our study the labyrinth was involved, which might have an influence on realization of total eradication depending on surgical circumstances. Lastly, less than half of our cases were primary surgeries, indicating that the vast majority of the cohort had a severe or aggressive disease. The number of included revision surgeries is comparable to the studies of Hellingman and van der Toom (6, 15), while Westerberg included primary cases alone, therefore these results are less comparable with our cohort (16).

Depending on the year of surgery, some cases had a longer follow-up with additional MRI-DWI(s) and outpatient clinical visits, up to five years. This could be a potential source of bias, which we have tried to address by also representing the data cumulative per year (Figure 1).

### 4.2 STAMCO classification

In our study each cholesteatoma was staged according to the STAMCO classification to report the data in a uniform way, making it easier to compare outcomes to other reports in the literature (19). In the stage III cholesteatomas, which comprise most of the cases, a significant improvement was shown in recidivism free rate when an obliteration was performed. The small numbers in the stage I group might explain why there was no significant difference between the obliteration and non-obliteration group.

### 4.3 Hearing outcome

To obliterate the epitympanic space the head of the malleus and incus need to be removed, necessitating ossicular chain reconstruction and possibly resulting in decreased conductive hearing ability. Surprisingly, the presented results prove otherwise: the postoperative hearing outcomes between both surgical techniques do not differ significantly. Cases with sensorineural hearing loss were evenly distributed over the two groups (Figure 2). Furthermore, the ABG closure 6 weeks postoperatively did not significantly differ between both groups (mean difference 1.5 dB in the obliteration group and 2.3 in the non-obliteration group, *p* = 0.903). However, it must be taken into account that our cohort mainly included large cholesteatomas (STAMCO stage II, III). For small cholesteatomas, where the ossicular chain is still functioning and intact, obliterating the epitympanic area could negatively affect the hearing since the ossicular chain then has to be removed. Further studies are necessary to evaluate this. Our main philosophical strategy is the preservation of structure and function where it is possible. Opting for highest odds of minimal disease recurrence (mastoid cavity and epitympanic area obliteration with ossicular chain reconstruction) vs. preserving the anatomy, is a shared decision process made together with the patient.

Limited literature exists regarding the hearing outcomes in patients treated with CWU followed by bony obliteration. Most research has been investigating hearing outcomes after CWD or canal wall reconstruction tympanomastoidectomy followed by bony obliteration, after which hearing seems to improve (10–13). A recent article did show improved hearing one year postoperatively after CWU in combination with obliteration, though the patients included were mostly treated for primary cholesteatoma (16). Our results, which showed no significant deterioration nor improvement for both techniques, may also be attributed to the fact that the majority of the cases were non-primary and extensive cholesteatomas (STAMCO stage II or III). Additionally, many cases had a history of other ear surgeries, leading to the prior removal of parts of the ossicular chain and sclerosis or fibrosis of the middle ear or tympanic membrane. Therefore, it can be inferred that the condition of the middle ear and the number of prior ear surgeries in the operated ear are likely the most significant predictive factors for hearing recovery.

### 4.4 MRI-DWI

Our study shows that an MRI-DWI one year postoperatively is a good tool to detect residual disease, but is not 100% accurate. In 22.5% (non-obliteration) and 1.4% (obliteration) of the cases the initial MRI-DWI did not show diffusion restriction, but after two or more years there turned out to be a cholesteatoma. In most of the cases a new retraction pocket had formed, indicating recurrent disease. However, in 4 cases (non-obliteration) a residual cholesteatoma was detected on the second MRI-DWI. This might be explained by the detection threshold and resolution power of the functioning MRI: disease smaller than 2 mm diameter is mostly not visualized on MRI-DWI. There were no false-negative residual cholesteatomas in the obliteration group. This also corresponds with the lower overall residual rate in the obliteration group. MRI-DWI has clear advantages compared to traditional second-look surgery (18, 30). It avoids the inherent operative risks and patient burden of surgery while simultaneously saving costs (31). In order to prevent undetected recurrent or residual cholesteatomas due to false-negative MRI-DWIs, we suggest that another sequential MRI scan is performed three and even five years postoperatively. This mentioned follow-up time is supported by Moller et al.: most cases with recurrent or residual disease appear within five years after the initial surgery (32).

## 5 Limitations and future perspectives

An important limitation of this study was the lack of randomization and its retrospective character. The location and extent of the cholesteatoma as well as the preference of the surgeon may have influenced both the choice for the surgical approach and the risk of recurrent and residual cholesteatoma. In addition, the follow-up time of the non-obliteration group was significantly longer compared to the obliteration group, making comparison between groups and drawn conclusions less robust. Some cases showed diffusion restriction on the MRI-DWI one year after surgery, that however disappeared on a second or third MRI-DWI. These false-positive results can only be exposed after an additional MRI-DWI, which may have influenced disease control in favor of the non-obliteration group. Lastly, the distribution between the STAMCO stages (I, II and III) varies largely within this cohort and it would be valuable to investigate the occurrence of recidivism within a more balanced group. It would therefore be useful to conduct a prospective, randomized controlled trial, to investigate the extent of the cholesteatoma and biology of the middle ear and look at possible correlations between disease, complaints, recidivism rates and hearing.

## 6 Conclusion

From this retrospective cohort study it is concluded that the bony obliteration technique of the epitympanic area and mastoid cavity after CWU tympanomastoidectomy improves disease control of cholesteatoma compared to no obliteration. Additionally, there is no significant difference in either technique with regard to the postoperative hearing outcome. To discover recurrent or residual disease another sequential three- and five year MRI-DWI is advocated. Future studies are necessary to correlate recidivism rates and hearing with the extent of the cholesteatoma and biology of the middle ear.

## Data Availability

The raw data supporting the conclusions of this article will be made available by the authors, without undue reservation.
