# Supplementary material for: Canal-wall up cholesteatoma surgery with mastoid obliteration leads to lower rates of disease recurrence without affecting hearing outcomes
Source: Front Surg. 2024 Apr 8;11:1381481. doi: 10.3389/fsurg.2024.1381481 (PMC11033303; doi:10.3389/fsurg.2024.1381481)
Supplement: Supplementary file 1 [file Datasheet1.pdf]

Supplementary files

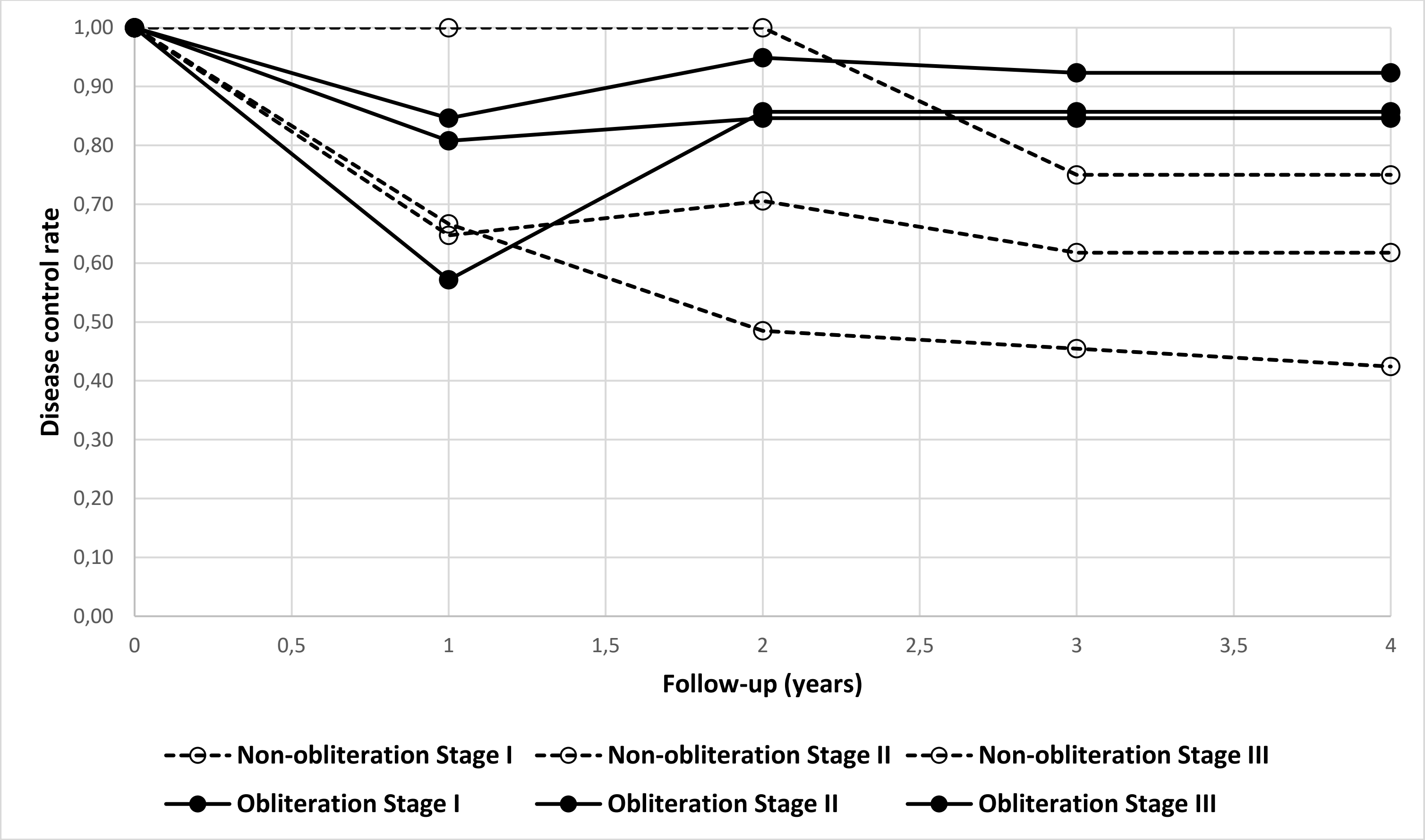

Supplementary file 1. Recurrent and residual disease rates divided by STAMCO stage: non-obliteration versus obliteration group.
The striped lines represent the non-obliteration group; the black solid lines represent the obliteration group. Per line the STAMCO stage is indicated in the graph. On the x-axis the follow-up time in years, on the y-axis the disease control rate (a disease control rate of 1 represents 0% recurrent or residual cholesteatoma cases).

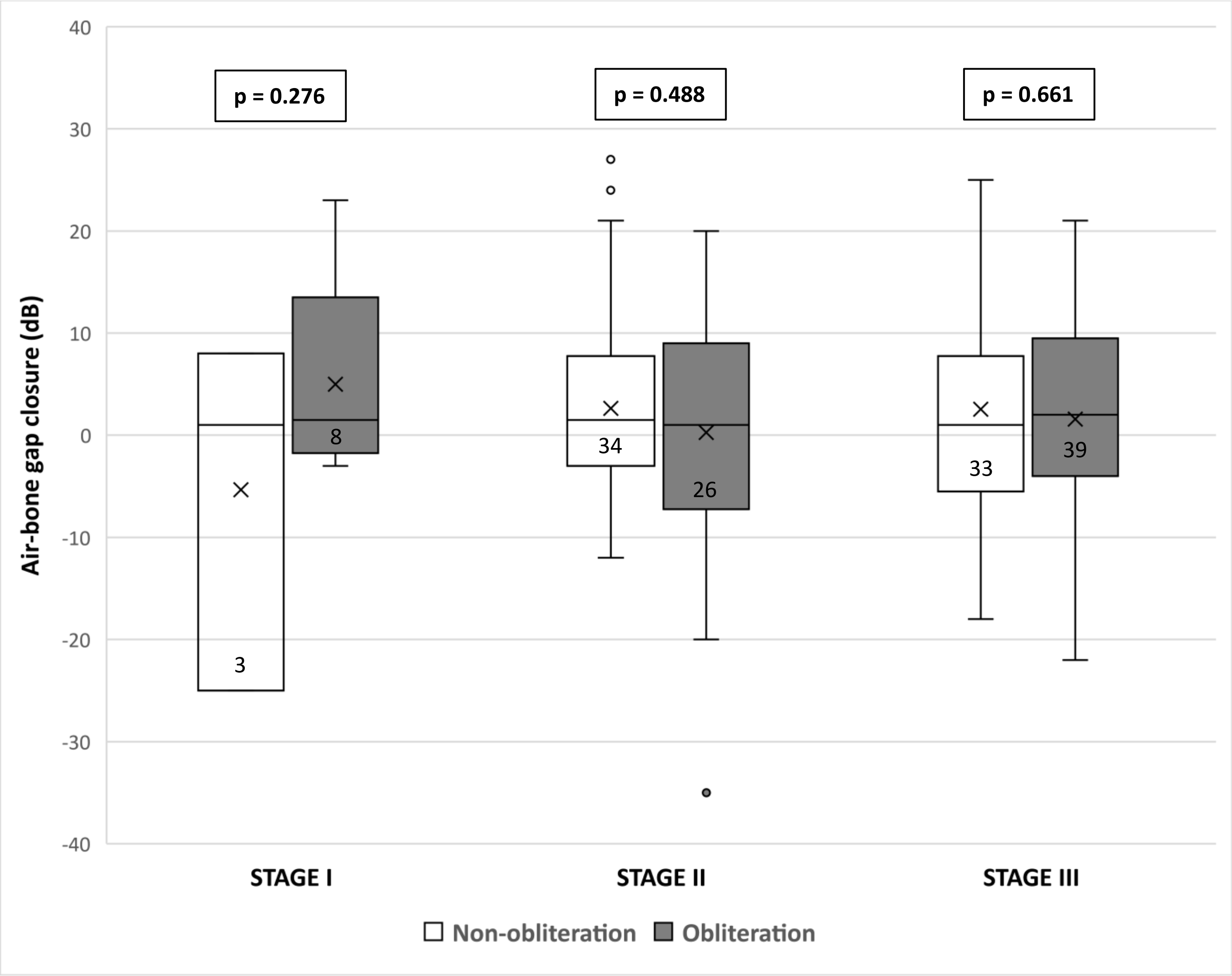

Supplementary file 2. Mean air-bone gap closure after 6 weeks divided by STAMCO stage (I,II,III): non-obliteration versus obliteration group.
The X in each box represent the mean. The number in each box represent the number of cases in that group.
